# A novel anti-pruritic: Topical co-administration of high molecular weight hyaluronan (HMWH) with protamine, a transdermal transport enhancer

**DOI:** 10.1177/17448069241254455

**Published:** 2024-05-10

**Authors:** Paul G Green, Jon D Levine

**Affiliations:** 1 Department of Oral & Maxillofacial Surgery, and UCSF Pain and Addiction Research Center, 8785 University of California at San Francisco, San Francisco, CA, USA; 2 Department of Preventative & Restorative Dental Sciences, and Division of Neuroscience, 8785 University of California at San Francisco, San Francisco, CA, USA; 3 Department of Medicine, and Division of Neuroscience, 8785 University of California at San Francisco, San Francisco, CA, USA

**Keywords:** Hyaluronan, itch, topical anti-pruritic, transdermal transporter, 5-hydroxytryptamine

## Abstract

Pruritis, the sensation of itch, is produced by multiple substances, exogenous and endogenous, that sensitizes specialized sensory neurons (pruriceptors and pruri-nociceptors). Unfortunately, many patients with acute and chronic pruritis obtain only partial relief when treated with currently available treatment modalities. We recently demonstrated that the topical application of high molecular weight hyaluronan (HMWH), when combined with vehicles containing transdermal transport enhancers, produce potent long-lasting reversal of nociceptor sensitization associated with inflammatory and neuropathic pain. In the present experiments we tested the hypothesis that the topical formulation of HMWH with protamine, a transdermal transport enhancer, can also attenuate pruritis. We report that this topical formulation of HMWH markedly attenuates scratching behavior at the nape of the neck induced by serotonin (5-hydroxytryptamine, 5-HT), in male and female rats. Our results support the hypothesis that topical HMWH in a transdermal transport enhancer vehicle is a strong anti-pruritic.

## Introduction

Pruritus (itch) is the most common symptom in dermatological disorders.^1,2^ Highly distressing, pruritis substantially impacts quality of life in patients with a variety of acute and chronic conditions and continues to pose a therapeutic challenge. Like pain, itch is generated sensitization of (itch) sensory neurons (pruriceptors).^3–5^ Of note, some pruriceptors are also nociceptors (prurinociceptors)^6,7^ and several pruritogens (e.g., 5-hydroxytryptamine (5-HT, serotonin), histamine, β-alanine, chloroquine) can also sensitize nociceptors, and produce hyperalgesia.^8–12^ Recently, we have shown that the topical application of high molecular weight hyaluronan (HMWH), in a vehicle containing transdermal transport enhancers (e.g., DMSO, protamine and terpenes), attenuates nociceptor sensitization associated with inflammation and painful peripheral neuropathies.^13^ In the present experiments we tested the hypothesis that our novel topical formulation of HMWH also attenuates pruritis produced by an exogenously administered pruritogen, 5-HT.^14–16^ We report that topical co-administration of HMWH with protamine, a transdermal transport enhancer,^17–19^ robustly attenuated scratching behavior induced by intradermal injection of 5-HT, at the nape of the neck, a well-established preclinical model of acute pruritis.^20^ And, as sex differences in pruritis have been described,^14,21,22^ we studied the anti-pruritic effects of topical HMWH in both male and female rats.

## Methods

### Animals

The present experiments were performed on 220–420 g adult male and female Sprague Dawley rats (Charles River Laboratories, Hollister, CA, USA). Experimental animals were housed three per cage, under a 12-h light/dark cycle, in a temperature- and humidity-controlled animal care facility at the University of California, San Francisco. Food and water were available in home cages, ad libitum. Testing of pruritogens was performed between 9:00 A.M. and 5:00 P.M.. Experimental protocols were approved by the Institutional Animal Care and Use Committee at the University of California, San Francisco, and adhered to the National Institutes of Health *Guide for the care and use of laboratory animals*. Effort was made to minimize the number of animals used and their suffering.

### Topical hyaluronan administration

5-HT (200 µg/10 µl) injected intradermally (i.d.) at the nape of the neck elicits acute scratching behavior that lasts more than 40 min. This acute scratching behavior elicited by intradermal 5-HT, was quantified by the method of Nojima and Carstens.^20^ Rats were acclimated to the testing chamber (24 × 45 cm) for 30 min, and then briefly anesthetized with 2% isoflurane, during which time the fur over the nape of the neck was shaved and 5-HT (200 µg in 10 µl 0.9% saline vehicle) injected, intradermally using a 30 gauge hypodermic needle.

**Figure 1.**
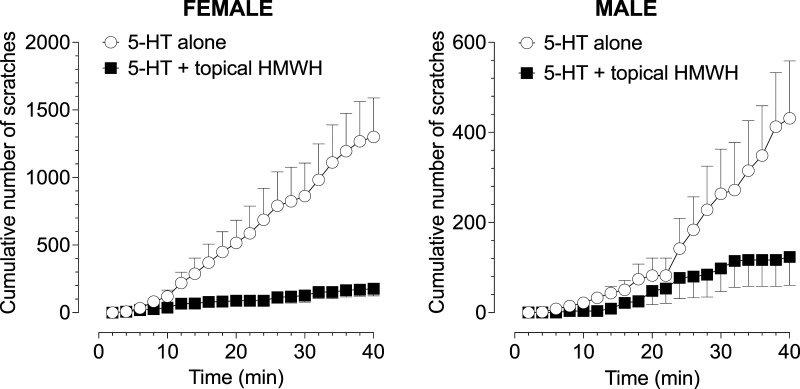
Topical HMWH attenuates 5-HT-induced scratching behavior in male and female rats. Rats received an intradermal (i.d.) injection of 5-HT, alone or after topically administered HMWH dissolved in protamine-containing vehicle. Data are presented here as cumulative number of scratches. Topical hyaluronan in a transdermal transport enhancer vehicle, protamine, markedly attenuated the number of 5-HT-induced scratches in both female and male rats (one-tailed paired Student’s t-test, females: *p* < .0001; males: *p* < .0004); all groups *n* = 6.

The high molecular weight hyaluronan was dissolved in protamine vehicle; protamine was first dissolved in distilled water (dH_2_O), to a concentration of 5 μg/μL, and stock solution of hyaluronan was combined with protamine with a final concentration of HMWH 2 μg/μL, in a volume of 30 μL. HMWH (30 μL) was administered topically on the nape of the neck, dispensed from a P200 pipette (Gilson, Middleton, WI, USA) with a plastic pipette tip, and then spread manually. In the topical HMWH group, HMWH was applied to the skin 30 min and again 5 min before the intradermal administration of 5-HT. Rats were then placed back in the testing chamber and their behavior video recorded for 40 min. Number of hind paw scratches directed at the 5-HT injection site were counted during video playback at half the recording speed. The total number of scratches in 2-min intervals is here presented (Figure 1).

### Drugs and their administration

The following drugs were used in the present experiments: 5-hydroxytryptamine (5-HT hydrochloride, Sigma, St. Louis, MO, USA); 500-1200 kDa high molecular weight hyaluronan (HMWH) purchased from Tocris (Minneapolis, MN, USA); and protamine sulfate purchased from Thermo Fisher Scientific (Waltham, MA, USA). HMWH was initially dissolved in dH_2_O at a concentration of 10 μg/μL, the stock solution, and further diluted by adding protamine. In the experiments in which hyaluronan was dissolved in protamine vehicle, protamine was first dissolved in dH_2_O, to a concentration of 5 μg/μL, and hyaluronan stock solution combined with protamine at a final concentration of 2 μg/μL, in a volume of 30 μL, for topical administration.

### Data analysis

Rats were treated with 5-HT alone, and again 4 days later with 5-HT after topical HMWH in protamine vehicle. Data are presented as cumulative number of scratches over 40 min (Figure 1). Experiments were performed with the experimenter blinded to experimental groups. Prism 10 (GraphPad Software) was used to generate graphics and to perform paired two-tailed t-test analyses; *p* < .05 is considered statistically significant. Data are presented as mean ± SEM.

## Results

In the current experiment we co-administered HMWH with the transdermal transport enhancer, protamine, to evaluate its effect on itch behavior elicited by intradermal 5-HT, a pruritogen that induces itch via action at 5-HT2 receptors,^23–27^ a receptor expressed on peptidergic and non-peptidergic sensory neurons,^23,28^ to test the hypothesis that topical HMWH attenuates 5-HT-induced scratching behavior. In both male and female rats, following topical HMWH treatment there was a significant attenuation in the total number of 5-HT-induced scratches (Males: 431.3 vs 123.3 scratches, *p = .0277, one-tailed t-test t(10) = 2.167; females: 1301 vs 176.8 scratches, **p = .0016 one-tailed t-test t(10) = 3.837) (Figure 1).

## Discussion

Itch (Pruritis), the major symptom of skin diseases^1,2^ which is experienced by ∼20% of the general population at some time,^29^ profoundly reduces quality of life.^2^ Like pain, itch is signaled by sensory neurons (pruriceptors), including prurinociceptors,^6,7^ and many pruritogens can also sensitize nociceptors.^22^ We previously found that topical HMWH inhibits inflammatory and neuropathic pain.^13^ In the present experiments we found that topically applied hyaluronan, when co-administered with a transdermal transport enhancer, protamine, markedly reduces 5-HT-induced scratching behavior in both male and female rats. These findings support the suggestion that our topical formulation of HMWH as a novel anti-pruritic therapeutic modality. These findings are consistent with our previous research demonstrating that topical application of hyaluronan, in a protamine vehicle, attenuated mechanical hyperalgesia induced by the pronociceptive inflammatory mediator prostaglandin E_2_, as well as that associated with chemotherapy-induced peripheral neuropathy, in rats receiving oxaliplatin or paclitaxel.^13^ The robust attenuation of itch-related behavior (i.e., scratching at the nape of the neck) induced by 5-HT, observed in both male and female rats, supports the suggestion that HMWH may have applicability for the management of pruritis. However, additional studies will be required to establish the broader applicability of topical HMWH to treat acute and chronic pruritis of diverse etiology.

## Conclusion

In summary, topical HMWH co-administered with a transdermal transport enhancer, protamine, markedly attenuated scratching behavior in a well-established preclinical model of acute pruritis, in which activity in sensory neurons (pruriceptors/prurinociceptors) provides the itch signal. Our findings support the use of topical HMWH, in a vehicle containing a transdermal transport enhancer, in the management of pruritis, of peripheral origin, as well as inflammatory and neuropathic pain, as shown previously,^13^ further underscoring its potential clinical utility.
